# Estrogenic activity and toxicity screening of Damnacanthal nanospheres and their metabolites assessed using an *in vitro* bioluminescent yeast assay

**DOI:** 10.1016/j.toxrep.2022.08.009

**Published:** 2022-08-22

**Authors:** Nutsawan Chaisutatip, Pleumchitt Rojanapanthu, Worapapar Treesuppharat, Thararat Nualsanit

**Affiliations:** a Department of Pharmacy, Faculty of Pharmacy, Mahidol University, Bangkok 10400, Thailand; b Drug Discovery and Development Center, Office of Advanced Science and Technology, Thammasat University, Pathum Thani 12120, Thailand; c Thammasat University Research Unit in Mechanisms of Drug Action and Molecular Imaging, Drug Discovery and Development Center, Office of Advanced Science and Technology, Thammasat University, Pathum Thani 12120, Thailand; d Chulabhorn International College of Medicine, Thammasat University, Pathum Thani 12120, Thailand

**Keywords:** Damnacanthal, Amphiphilic chitosan nanospheres, Bioluminescent yeast assay, Estrogenic activity, Metabolic activation

## Abstract

The root of *Morinda citriforia* L. (Noni) was used to extract Damnacanthal (Damna), an anthraquinone compound. In this study, Damna was successfully incorporated in *N*-phthaloylchitosan-grafted poly (ethylene glycol) methyl ether (PhCS-g-mPEG) to form Damna nanospheres (Damna-NS) with the particle size 298 nm and the incorporation efficiency 36.30 %. A bioluminescent yeast-reporter system was used to assess Damna-NS’s estrogenic or toxic effects. The initial screening results revealed that both Damna and Damna-NS themselves showed no estrogenic effect. They showed strong effects when treated with a S9 fraction or liver microsomes, showing that their metabolites are estrogenic. Toxicity tests demonstrated that Damna and Damna-NS are harmful when used alone; however, they showed no toxicity when treated with S9 mix. In conclusion, the findings showed that Damna-NS, when taken as an oral phytoestrogen for hormone replacement treatment, has the potential to endanger human health by producing estrogenic effects and minimizing harmful effects in the liver.

## Highlights

• Damnacanthal can be incorporated in PLC-g-mPEG polymer to form nanospheres.
• Bioluminescent yeast-reporter system can investigate damnacanthal nanosphere’s estrogenic or toxic effects.
• Damnacanthal nanospheres provide a promising benefit as an oral phytoestrogen in hormone replacement therapy.

## Graphical Abstract

**Figure:**
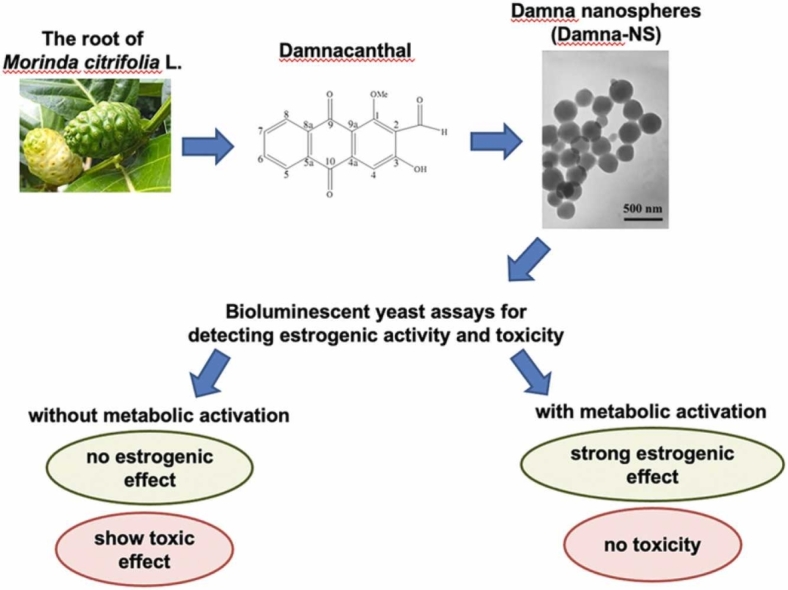

## 1 Introduction

*Morinda citrifolia* L*.* (Rubiaceae) is a traditional Thai folk medicinal plant known as "noni" or "Yor". It has been used for a variety of medical reasons in Polynesia and as a plant-based medicine in Thailand and other Southeast Asian nations. Noni has been shown to provide a wide range of health advantages, including anti-cancer, anti-infection, anti-diabetes, anti-asthma, anti- hypertension, anti-inflammatory properties, anti-hemorrhoids and anti-rheumatoid arthritis [1], [2].

Anthraquinone compound damnacanthal (Damna) was derived from the roots of *Morinda citrifolia* L. and discovered as a strong inhibitor of p56^lck^ tyrosine kinase activity through high-volume screening of natural product extracts [3]. Damna also exhibits potent anti-cancer activity [3], [4], [5] and anti-inflammation activity [4], [6], [7]. In addition, Damna has antifungal and antituberculosis properties against *Candida albicans* and *Mycobacterium tuberculosis*, respectively [4].

Despite Damna's significant potential for preserving and boosting health advantages, its low solubility and poor stability have reduced its bioavailability and target specificity, which, when combined with the side effects reported when administered at high doses, has limited its use. Therefore, for the past few decades many attempts have been made to solve these problems. Drug delivery technologies such as liposomes, polymeric nanoparticles, and lipid nanoparticles have sparked a lot of interest in the field of nanotechnology (SLN, NLC). These drug delivery systems were created to change and improve the pharmacokinetic and pharmacodynamic aspects of numerous therapeutic phytochemicals [8]. In recent years, self-assembled polymeric micelles have emerged as an appealing drug delivery mechanism among these options. Amphiphilic block or graft copolymers can generate a unique nanosized core-shell structure with a hydrophobic core as a reservoir for water-insoluble medicines and a hydrophilic shell to boost stability and prolong circulation, lowering reticuloendothelial system uptake (RES) [9], [10], [11].

Estrogen (or oestrogen) is a steroid hormone that is produced mostly by the ovaries and is involved in the development and maintenance of female sexual characteristics. Estrogen also has a number of pharmacological roles, including bone mass maintenance, cardiovascular protection, and brain protection [12], [13]. Hot flushes, sleeping disturbances, vaginal dryness, joint discomfort, mood changes, and diminished bone density are among symptoms of estrogen insufficiency during menopause [14], [15], [16]. Phytoestrogens, a new class of medicinal compounds derived from plants that exhibit estrogen-like activity [17], can be used to treat menopausal symptoms with minimal adverse effects [17], [18].

The bioluminescent *Saccharomyces cerevisiae* strains BLYES and BLYR were created to detect the presence of estrogenic and poisonous chemicals in the environment, respectively. The bioluminescent *luxCDABE* genes from *Photorhabdus luminescens* and the *frp* gene from *Vibrio harveyi* are expressed in two plasmids, either under the control of estrogen response elements (*S. cerevisiae* BLYES) or constitutively, for toxicity detection (*S. cerevisiae* BLYR). A bioassay based on these bioreporter strains has been shown to be a high-throughput, low-cost, and quick screening technique for identifying possible endrocrine disrupting compounds (EDCs) [19], [20].

To our knowledge, no research has been published on the synthesis of Damna nanospheres (Damna-NS) in *N*-phthaloylchitosan-grafted poly (ethylene glycol) methyl ether (PhCS-g-mPEG). We offer a simple self-assembled approach for synthesizing Damna-NS with a controllable size and drug delivery characteristic in this paper. The morphology, size, formation conditions, drug loading and *in vitro* drug release profile of the nanospheres were studied. We applied bioluminescent yeast reporter to screen the estrogenic property and toxicity of Damna-NS. We also investigated how the metabolism of test samples by human liver S9 fraction affects their estrogenic property and toxicity.

## 2 Materials and methods

### 2.1 Materials

Biomedical grade chitosan (average molecular weight of 5,000 kDa, degree of deacetylation 95 %) was supplied by Seafresh Chitosan (Lab) Company (Thailand). Phthalic anhydride and succinic anhydride were purchased from Fluka Chemical Company (Switzerland). Poly (ethylene glycol) methyl ethers (mPEG, average molecular weight of 5,000 Da) were obtained from Aldrich Chemical Company (USA). 1-Ethyl-3-(3 ´-dimethylaminopropyl)carbodiimide (EDC) was purchased from Tokyo Chemical Industry (Japan). 1-Hydroxybenzotriazole monohydrate (HOBt) was obtained from BDH Laboratory Supplies (UK). Dichloromethane and hexane were purchased from Samchai Chemical Company (Thailand). Ethyl acetate, chloroform, dimethylsulfoxide (DMSO), dimethylformamide (DMF), ethanol and methanol were purchased from Lab Scan Analytical Sciences (Thailand). Potassium dihydrogen phosphate (KH_2_PO_4_) was obtained from Ajax Finechem (Australia). All other reagents were of analytical grade and deionized (DI) water was used throughout the study.

### 2.2 Damnacanthal

As previously reported, Damna was isolated from the roots of *Morinda citrifolia* L. in Thailand [21]. Various approaches were used to confirm the isolated Damna, including thin layer chromatography (TLC), melting point, liquid chromatography–mass spectrometry (LC-MS), and ^1^H and ^13^C nuclear magnetic resonance spectroscopy (NMR).

### 2.3 Amphiphilic chitosan nanospheres

Amphiphilic chitosan nanospheres were made as described before [22], [23], [24]. The following is a brief description of the preparation process.

#### 2.3.1 *N*-phthaloylchitosan (PhCS) synthesis

Chitosan (1 g) was reacted for 6 h at 100 ºC under reduced pressure with phthalic anhydride (4.48 g, 5 mol equivalent to pyranose ring) in DMF (20 mL). The mixture was stirred overnight at 60 ºC. PhCS was obtained by reprecipitating the combination in an ice bath, extensively washing with ethanol for three times using a centrifugation at 12,000 rpm for 10 min to remove the unreacted components followed by drying at room temperature under vacuum.

#### 2.3.2 mPEG terminated with carboxyl group (mPEG-COOH) synthesis

Succinic anhydride (1 mol equivalent to mPEG) was reacted with mPEG at 60 ºC overnight in DMF in the presence of the catalytic amount (pyridine). mPEG-COOH was obtained by reprecipitating the mixture in diethyl ether and drying at room temperature under vacuum.

#### 2.3.3 PhCS-g-mPEG synthesis

In 15 mL of DMF solution containing HOBt and EDC (3 mol equivalent to mPEG-COOH), the mPEG-COOH (0.40 mol) was agitated overnight with PhCS (3.71 × 10^−3^ mol) at room temperature. To get white particles of PhCS-g-mPEG, the mixture was dialyzed in DI water and extensively washed in methanol.

### 2.4 Damna incorporation into amphiphilic chitosan nanospheres

Damna was incorporated into polymeric micelles to form Damna-NS via a dialysis procedure [25]. PhCS-g-mPEG polymer and Damna (5–60 % w/w of the polymer) were dissolved in 5 mL DMSO and kept out of the light. To allow the drug to incubate with the polymer, the mixture was swirled at room temperature for 24 h. The mixture was subsequently dialyzed against DI water overnight in a dialysis bag (Spectra/Por® 12,000–14,000 MWCO, Spectrum Laboratories, USA). The DI water was changed every day until the amount of DMSO was lower than the limit of detection (0.5 mg/L) [26]. A milky solution was obtained and stored at 4 ºC. HPLC (Shimadzu, Japan) system equipped with a pump (LC-10AT VP), a controller (SCL-10A VP) and a UV detector (SPD-10A VP) at 282 nm was used to evaluate the amount of Damna incorporated into amphiphilic chitosan nanospheres. A synergi 4 µm, Max-RP 80 A 250 × 4.6 mm column (Phenomenex®, USA) was used with a mobile phase of methanol: 0.02 M KH_2_PO_4_ (70:30) at pH 3 and a flow rate of 1 mL/min. The encapsulation efficiency (EE) of Damna was calculated as Eq. (1).

(1) EE (%) = [W_*d*_/W_*o*_] × 100

where W_*d*_ is the amount of Damna in nanospheres and W_*o*_ is the initial amount of Damna.

### 2.5 Characterization of Damna-NS and blank nanospheres (Blank-NS)

#### 2.5.1 Particle size, polydispersity index, and zeta potential measurements

The prepared Damna-NS suspension was measured its particle size and polydispersity index by dynamic light scattering (DLS) technique using a Coulter submicron particle analyzer (N4MD, Coulter Electronics, USA) and its zeta potential by electrophoretic mobility using a Zetasizer (Nano-ZS, Malvern, UK). The sample was diluted with DI water at the ratio of 1:6 to achieve a suitable concentration before measurement.

#### 2.5.2 Thermal analysis

To analyze thermal properties of Damna, Damna-NS, and Blank-NS, the differential scanning calorimetry (DSC) were performed using a DSC821e apparatus (Mettler Toledo, Switzerland). The measurements were carried out under nitrogen gas at a scanning rate of 20 °C/min in the temperature range of 30–300 °C. The melting point, melting enthalpy (∆H), and onset temperature were evaluated using the STARe Software (Mettler Toledo, Switzerland).

#### 2.5.3 Transmission electron microscopy (TEM)

The morphology of Damna-NS was observed by a TEM (Hitachi H-2000 electron microscope, Japan). The Damna-NS was sonicated for 2 min, dropped onto paraffin, and then covered with a copper grid for 10 min. The samples were negatively stained with 2 % uranyl acetate used as a metal stain to enhance the contrast of samples and then air-dried prior to imaging.

#### 2.5.4 Fourier transform infrared spectrophotometry (FT-IR) analysis

The dried samples of Damna, Damna-NS, and Blank-NS were grounded and mixed with potassium bromide (KBr) followed by compressing into pellets. Each FT-IR spectrum was recorded on a 16PC FT-IR spectrophotometer (Perkin Elmer, Beaconsfield, England) with a wavenumber range of 4000–400 cm^−1^.

### 2.6 *In vitro* drug release studies

The dialysis bag diffusion technique was used in the in vitro drug release tests [27], [28], [29], [30]. For this, 8 mL of Damna-NS suspension was placed into a pre-swelled dialysis bag with a 12-kDa molecular weight cutoff (a pore size of 2.4 nm), which was subsequently immersed in 150 mL of PBS solution (as release medium) with pH 5.5 and 7.4. The drug release experiments were carried out at 37 °C with a steady vibration of 100 rpm. At the time intervals of 10, 30 min, 1, 2, 4, 8, 12, 24, 48, 72, 96, 120, 144, and 168 h, the release medium (50 mL) was removed and replaced with a fresh PBS solution in an equal volume. A calibration curve of Damna using a Shimadzu HPLC was used to determine the Damna concentration in the release medium. All tests were carried out in triplicate.

The results were expressed as a cumulative percentage release as Eq. (2).

(2) Cumulative release (%) = (W_*t*_ / W_*l*_) × 100

where W_*t*_ is the amount of Damna released from nanospheres at time *t* and W_*l*_ is the amount of Damna loaded into the nanospheres.

### 2.7 Growth of strains

The bioluminescent *Saccharomyces cerevisiae* yeast reporter strains BLYES and BLYR have been described previously [19], which were performed to detect the presence of estrogenic and toxic compounds, respectively. These yeast reporters were obtained from the Center for Environmental Biotechnology (CEB), The University of Tennessee, Knoxville, Tennessee, USA. The freezer stocks of *S. cerevisiae* BLYES and *S. cerevisiae* BLYR were thawed and grown in 25 mL of the yeast minimal media (YMM leu^-^, ura^-^) at 30 °C overnight with constant shaking at 200 rpm. When an optical density at 600 nm was about 1.0, these yeast cells were placed into black 96-well Microfluor microtiter plates for further assay.

### 2.8 Bioluminescent yeast assays for detecting estrogenic activity and toxicity of Damna-NS

Two microliters of a serial dilution of Damna in DMSO (1 ×10^−2^ – 2.5 ×10^−8^ M), Damna-NS in DI water (2 ×10^−2^ – 5 ×10^−8^ M), and Blank-NS in DMSO (1 ×10 ^0^ – 2.5 ×10^−6^ M) were added to a black 96-well plate. Equal volume (100 µL) of DI water and yeast strains (BLYES and BLYR for estrogenic activity and cytotoxicity assay, respectively) were added into each well of the black 96-well plate. All samples were performed in triplicate. Bioluminescence was measured every 60 min for 5 h in a Perkin-Elmer Victor^2^ Multilabel Counter with an integration time of 1 s per well. 17β-estradiol (1 ×10^−7^ – 2.5 ×10^−13^ M) was used as a positive control for the estrogenic activity assay. DMSO mixed with DI water was used as a negative control.

### 2.9 Bioluminescent yeast assays for in vitro studies in drug metabolism of Damna-NS using human liver S9 fraction

Two microliters of a serial dilution of Damna in DMSO (1 ×10^−2^ – 2.5 ×10^−8^ M), Damna-NS in DI water (2 ×10^−2^ – 5 ×10^−8^ M), and Blank-NS in DMSO (1 ×10 ^0^ – 2.5 ×10^−6^ M) were mixed with 12 µL of 20 mM NADPH-regenerating system in PBS solution pH 7.4 and 5 µL of 0.5 mg/mL of liver S9 fraction and were then added to 100 mM PBS solution pH 7.4 to make the final volume up to 200 µL. The incubation was carried out at 37 °C for 60 min with gentle shaking. After 60 min of reaction, the reaction was terminated by adding 200 µL DMSO.

For in vitro drug metabolism assay, after incubation of test samples with liver S9 fractions, 2 µL of a serial dilution of incubated Damna in DMSO (1 ×10^−4^ – 2.5 ×10^−10^ M), incubated Damna-NS in DI water (1.4 ×10^−5^ – 3.5 ×10^−11^ M) and incubated Blank-NS in DMSO (2 ×10^−8^ – 5 ×10^−14^ M) were added into a black 96-well plate. Two hundred microliters of yeast strains (BLYES and BLYR) were added into each well of the black 96-well plate. All samples were performed in triplicate. Bioluminescence was measured every 60 min for 5 h in a Perkin-Elmer Victor^2^ Multilabel Counter with an integration time of 1 s per well. Incubated 17β-estradiol (1 ×10^−4^ – 2.5 ×10^−10^ M) was used as a positive control for the estrogenic activity assay. Vehicle (DMSO or DI water) was used as a negative control.

### 2.10 Bioluminescent yeast data analysis

Bioluminescence (counts per sec) was plotted against the log of chemical concentration (M) for each test sample, resulting in a sigmoidal curve for hormonally active chemicals. The midpoint of the linear section of the sigmoidal dose-response curve was used to calculate the 50 % effective concentration (EC_50_). To quantify the variability between assays, the mean and standard deviation values were derived from repeated EC_50_ values for each test sample. Toxic reactions (IC_20_) were calculated by dividing the chemical concentration by the baseline bioluminescence by 20 %.

### 2.11 Analytical statistics

The data is presented as a mean ± SD. The *t*-test was used to make comparisons between two means. ANOVA techniques were used to perform analysis of variance in IBM SPSS Statistics software, version 21 (IBM Corp., Armonk, NY, USA). A *p* value < 0.05 was considered statistically significant.

## 3 Results and discussion

### 3.1 Factors affecting morphology, particle size and incorporation efficiency of Damna-NS

In this study, we looked into the impact of the amount of initial Damna in the formulations ranging from 5 % to 60 % on the incorporation efficiency and particle size of Damna-NS. The findings showed that the drug entrapment efficiency increased from 1.26 ± 1.77 % to 36.30 ± 8.96 % with increasing the ratio of Damna: Blank-NS in the formulations as shown in Table 1. Formulation at the ratio of Damna: Blank-NS, 1:5 (20 % initial Damna in the formulation) had the highest entrapment efficiency.

**Table 1:** The effect of the initial drug loading on the incorporation efficiency and the particle diameters of Damna-NS.

Initial Damna (% of polymer)	Damna: Blank-NS	% Encapsulation	Average particle size (nm)
0	0: 5	0	243.0 ± 2.5
5	0.25: 5	1.26 ± 1.77	248.6 ± 2.1
10	0.50: 5	8.76 ± 6.70	250.3 ± 1.6
15	0.75: 5	16.22 ± 9.21	274.7 ± 2.1
20	1.00: 5	36.30 ± 8.96	298.0 ± 2.7
25	1.25: 5	-a	330.0 ± 7.4^a^
30	1.50: 5	-a	395.7 ± 13.3^a^
40	2.00: 5	-a	412.2 ± 15.1^a^
50	2.50: 5	-a	1384.4 ± 190.9^a^
60	3.00: 5	-a	1024.8 ± 12.02^a^

a The precipitated crystal of excess drug as investigated by TEM

**Fig. 1:**
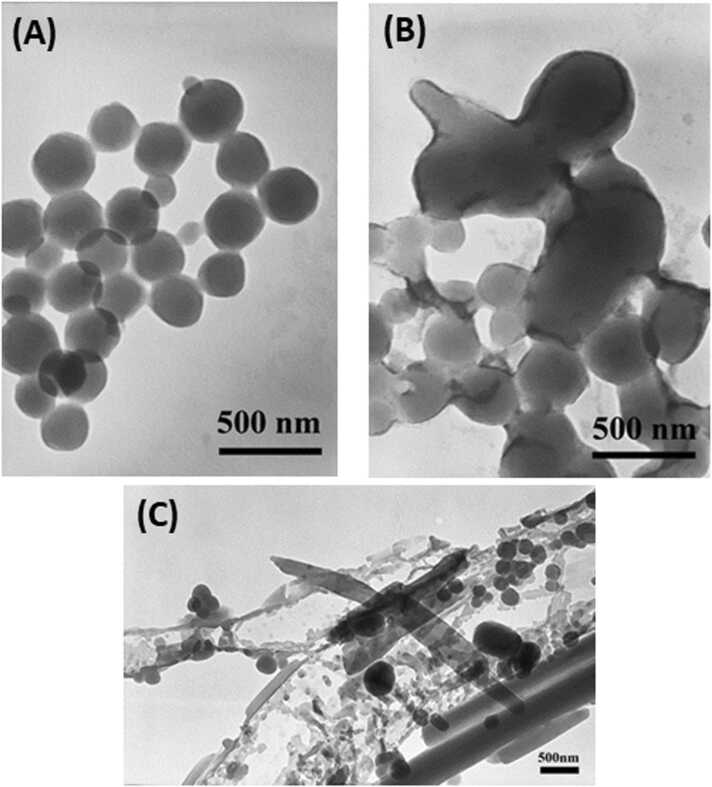
Transmission electron microscope of Damna-NS prepared under (A) the optimal conditions at pH 3.8, (B) the acid conditions (pH > 5.0), and (C) excess initial drug loadings at a magnification of (A and B) 80,000 × and (C) 30,000 × .

The 20 % initial drug loading Damna-NS colloidal suspension had a mean diameter of 298 ± 2.7 nm (Fig. 1A). An initial drug loading higher than 20 % resulted in the precipitated crystal of excess drug as investigated by TEM (Fig. 1C). We conclude that the optimum initial drug for use in Damna-NS preparation should be 20 %. The hydrophobic interactions between the hydrophobic PhCS chain, Damna, and the solvent were the most critical component in controlling this incorporation process. Micelle formation and drug incorporation into micelle were predicted to happen at the same time [31]. From the phenomenon of nanospheres, the hydrophobic phthalimido groups accumulated in the center and the hydrophilic mPEG chains interacting with water molecules outside the core caused the PhCS-g-mPEG to self-assemble in water. The Damna-NS were colloidal solution form after preparation that were composed of spherical and uniformly shaped nanoparticles. According to certain investigations, Damna was successfully created as chitosan-based nanoparticles utilizing chitosan that had been grafted with deoxycholic and poly(ethylene glycol) methyl ether (DCA-CS-mPEG) [32], [33].

To evaluate the appropriate pH value of the second-round acid dialysis medium in order to improve the morphology of nanospheres, we altered the pH value of the second-round dialysis medium from 2.6 to 5.4. The most suitable pH value of acid dialysis medium was found to be 3.8 in order to achieve spherical and uniform nanospheres, while avoiding stretched shapes. If the pH values of dialysis medium of less than 3.2 showed the ‘salting out’ phenomenon. If the pH values of the dialysis medium were more than 5.0, stretched and non-uniformly spherical nanospheres occurred (Fig. 1B). The zeta potential of Damna-NS dispersed in water was − 31.2 ± 5 mV as shown in Table 2, implying that the nanosphere was covered in negatively charged electrons. A previous study reported that following IV administration to rats, negatively charged nanoparticles cleared from the blood more slowly than positively charged nanoparticles and persisted in blood stream for a longer period of time [34]. Additionally, negatively charged materials demonstrated lower cytotoxicity than positively charged ones and approved by FDA [35]. Therefore, it is important to optimize the surface charge density of nanoparticles for minimal toxicity and efficient intracellular drug delivery. Furthermore, the zeta potential value of colloidal systems and nano-medicines, as well as their particle size have a significant impact on the different characteristics of nano-drug delivery systems since they influence particle stability as well as cell adhesion [36].

**Table 2:** Characteristic properties of Damna-NS.

Sample	Mean diameter (nm)^a^	Polydispersity Index^a^	Zeta potential (mV)^a^
Damna-NS	298.0 ± 2.7	0.288 ± 0.062	-31.2 ± 5

a Each value represents the mean ± SD (n = 3).

### 3.2 Thermal properties

**Fig. 2:**
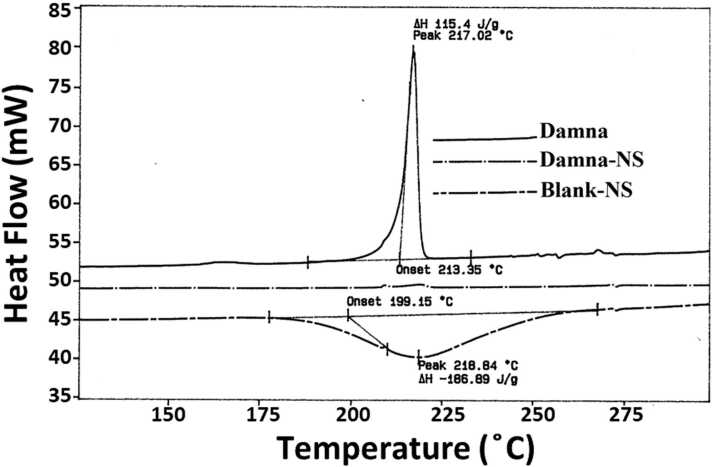
Differential scanning calorimetry (DSC) of Damna, Damna-NS and Blank-NS.

In order to determine whether a formulation is crystalline or amorphous, as well as to assess drug-polymer interactions, DSC experiments were conducted. DSC curves of Damna, Damna-NS and Blank-NS are presented in Fig. 2. The pure Damna showed a crystalline nature and demonstrated a sharp peak at 217.02 °C, which could be related to its melting point. Blank-NS showed an exothermic peak at 218.84 °C. In nanoparticle formulation, Damna-NS exhibited an amorphous nature and no endotherms were observed in its DSC thermogram. This showed the success of Damna entrapment in nanospheres.

### 3.3 FTIR analysis

**Fig. 3:**
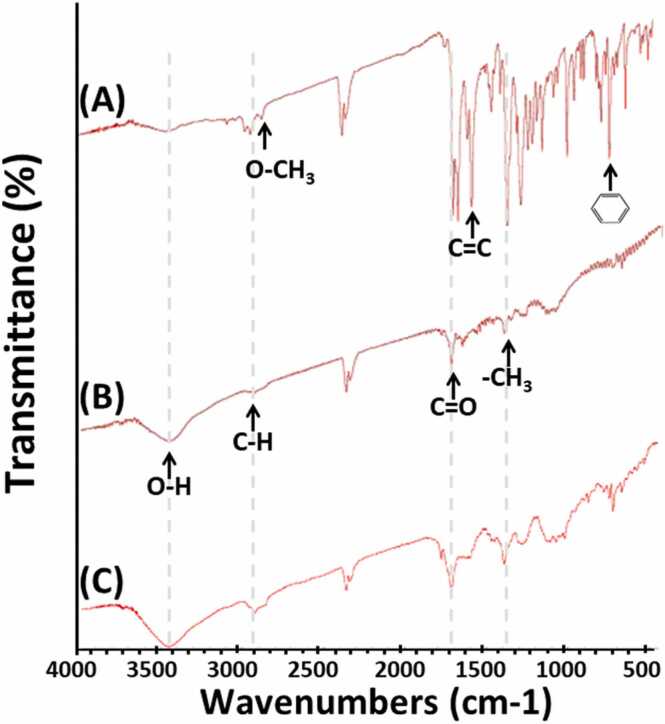
The FT-IR spectrum of (A) Damna, (B) Damna-NS and (C) Blank-NS.

FTIR analysis of Damna-NS was performed to investigate the interaction between Damna and the PhCS-g-mPEG polymer. Fig. 3 indicates the comparative FTIR peaks of Damna, Damna-NS and Blank-NS. As described in Fig. 3A, pure Damna has specific characteristics, such as absorption peaks at 719, 1348, 1564, 1649, 2855, 2946 and 3434 cm^−1^ corresponding to aromatic ring, methyl group, C=C stretching vibration, C=O stretching vibration, symmetric C-H stretching vibration, asymmetric C-H stretching vibration and O-H stretching vibration, respectively [37], [38], [39]. Fig. 3C shows the characteristic features of Blank-NS comprising the absorption peaks at 1345, 1716, 2874 and 3463 cm^−1^ corresponding to methyl group, C=O stretching vibration, C-H stretching vibration and O-H stretching vibration, respectively [22], [23]. For Damna-NS (see Fig. 3B), the distinctive absorption peak was most prominent at 1345, 1695, 2905 and 3443 cm^−1^ corresponding to methyl group, C=O stretching vibration, C-H stretching vibration and O-H stretching vibration, respectively. Freeze dried Damna-NS exhibited mainly the absorption peaks of Blank-NS overlapping with a few peaks of Damna. It can be concluded that there was no strong interaction between Damna and the polymer during the nanospheres formation.

### 3.4 *In vitro* drug release studies

**Fig. 4:**
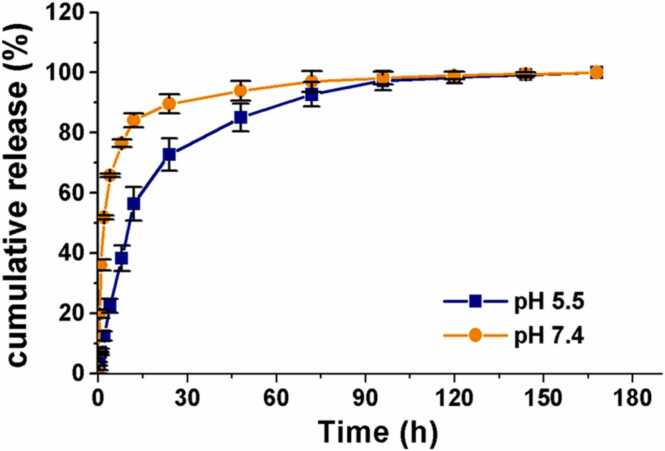
Cumulative release of Damna-NS at 37ºC in PBS solution under pH 5.5 and 7.4.

The in vitro drug release from Damna-NS at a ratio of Damna: Blank-NS (1:5) in different pH is demonstrated in Fig. 4. The release of Damna in PBS buffer pH 7.4 was burst during the first 12 h. Damna's release rate dropped after that and reached a stable level after 24 h. This burst stage can be attributed to the release of Damna absorbed at the region near or within the PEG shell of nanospheres and its ability to access the aqueous medium without the need for long-time diffusion. The steady release can be attributed to the releasing of Damna trapped in the core of the nanospheres.

The release profile of Damna in PBS buffer pH 5.5, which mimics the acidic microenvironment in endosomes/lysosomes, showed a gradual release of Damna in the first few hours of the initial stage. Fifty percent of the total drugs were released in first 12 h that were longer than in pH 7.4, followed by an extended- release period of more than 84 h.

In this investigation, the sustained release of Damna was clearly observed in pH 5.5.

Damna is a weak acidic drug with pKa value of 5.26 ± 0.2. The solubility of a weak acidic drug is strongly influenced by the pH of the solution. Changes in the pH of PBS buffer can alter the solubility of Damna. Damna will be more poorly soluble in an acidic medium (pH 5.5), while it will be more water-soluble in an alkaline solution (pH 7.4). This shows that the prepared Damna-NS have a good entrapping ability and could be developed as a controlled release drug carrier.

### 3.5 Bioluminescent yeast assays for detecting estrogenic activity and toxicity of Damna-NS

**Fig. 5:**
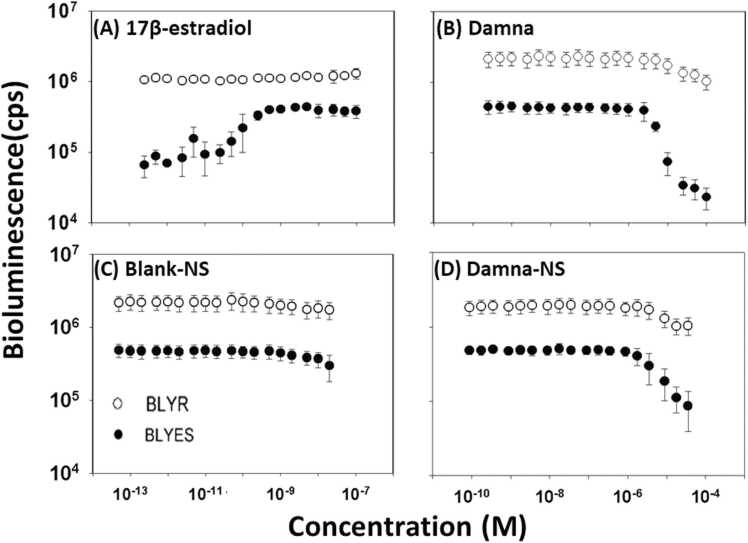
Dose-response curves of (A) 17β-estradiol, (B) Damna, (C) Blank-NS, and (D) Damna-NS using *S. cerevisiae* BLYES (closed circles) and *S. cerevisiae* BLYR (open circles) reporter strains. Data were obtained 4 h post-induction and expressed as counts per second (cps). Each value represents the means ± SD (n = 7).

*Saccharomyces cerevisiae* bioluminescent bioreporter assays*, S. cerevisiae* BLYES and *S. cerevisiae* BLYR were used to access a compound’s estrogenic or toxic effects, respectively. Chemicals that are hormonally active showed a sigmoidal curve in each assay. A dose-response curve for positive control, 17β-estradiol, using strain BLYES are shown in Fig. 5A. The 17β -estradiol displayed a full sigmoidal dose-response curve and the EC_50_ value was 1.07 × 10^−10^ M; there was no decrease in bioluminescence in the BLYR strain. The value is in agreement with previously published data [19], [20]. At high concentrations, Damna strain BLYES showed a dramatic decrease in bioluminescence, indicating chemical toxicity; the IC_20_ value was 1.28 × 10^−5^ M (Fig. 5B).

Blank-NS showed no increase in bioluminescence in the BLYES strain and no decrease in bioluminescence in the BLYR strain, indicating that it is neither hormonally active nor poisonous (Fig. 5C). In the BLYES strain, Damna-NS similarly demonstrated a dramatic drop in bioluminescence at high concentrations, with an IC_20_ value of 3.20 × 10^−5^ M (Fig. 5D), implying chemical toxicity. The reproducibility of the bioreporter assays was determined by calculating the IC_20_ of Damna and Damna-NS from seven independent experiments. The toxic effects of Damna and Damna-NS were confirmed with the constitutive bioreporter (BLYR).

### 3.6 Metabolic activation of Damna-NS by human liver S9 fraction

**Fig. 6:**
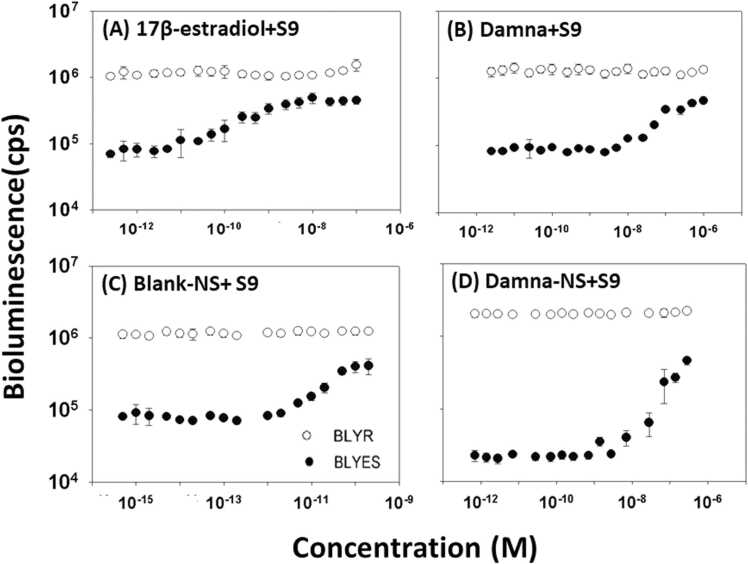
Dose-response curves of (A) 17β-estradiol, (B) Damna, (C) Blank-NS, and (D) Damna-NS incubated with human S9 fraction using *S. cerevisiae* BLYES (closed circles) and *S. cerevisiae* BLYR (open circles) reporter strains Data were obtained 4 h post-induction and expressed as counts per second (cps). Each value represents the means ± SD (n = 4).

For the estrogenic screening of metabolites, a metabolic activation system containing a S9 fraction or liver microsomes was applied to *Saccharomyces cerevisiae* bioluminescent bioreporter experiments in vitro. The response to metabolites of the positive control, 17β-estradiol, was investigated in the BLYES and BLYR strains (Fig. 6A). In the BLYES strain, the metabolite of 17β-estradiol formed a full sigmoidal dose-response curve with an EC_50_ value of 5.28 × 10^−10^ M and no decrease in bioluminescence. The metabolic activation patterns in BLYES with the S9 portion of Damna, Blank-NS, and Damna-NS displayed a sigmoidal curve similar to 17β-estradiol, as shown in Figs. 6B, 6C and 6D, indicating their metabolites to be estrogenic. The metabolites of Damna, Blank-NS and Damna-NS had the EC_50_ values of 1.32 × 10^−7^, 2.87 × 10^−11^ and 1.27 × 10^−7^ M, respectively. None of the test metabolites showed the toxic effect in BLYR strain (Figs. 6B, 6C and 6D). Damna and Damna-NS have no estrogenicity on their own, but when given a S9 fraction or liver microsomes, they had strong effects, showing that their metabolites are estrogenic.

Our results were in accordance with Yoshihara’s study that showed a metabolic activation of estrogenic activity of bisphenol A, a well-known endocrine-disrupting chemical in environment, by rat liver S9 fraction [40]. Damna and Damna-NS were shown to be toxic when tested individually; however, they demonstrated no toxicity when treated with the S9 fraction.

Generally, an in vitro bioluminescent yeast assay is typically used for screening the toxicity and estrogenicity of chemicals in the environment. In this study, we have applied this testing method in the field of medical research to determine the toxicity and the estrogenicity of biological compounds because of low cost, high-throughput, and rapid screening method. We are the first to use this assay to determine the estrogenic potential of Damna-NS. The metabolites of Damna-NS apparently exhibited an estrogenic property. This characteristic could result from Damna and/or chitosan components. Chearskul et al. reported that subcutaneous injection of *Morinda citrifolia* L. extract in mice for 3 consecutive days showed the estrogenic activity [41]. Damna is one of the major compounds in the root extract of *Morinda citrifolia* L., supposing that Damna should possess estrogenic properties. Wu et al. have shown in another study that the estrogen-deficient rats were fed with chitosan for 12 weeks alleviated menopausal symptoms by improving the variety and composition of the gut microbiota as well as serum metabolites [42]. These findings led us to the conclusion that Damna formulation in chitosan nanospheres would have a synergistic effect on the estrogenic action, which might lead to its use in estrogen hormone replacement therapy. However, further research is necessary to determine the chemical identification and structural elucidation of the unknown metabolites produced by Damna-NS, as well as the molecular mechanisms behind the influence of the unidentified metabolites on estrogenic activity.

## 4 Conclusion

The spherical and uniform shape of Damna-NS with a diameter of 298 ± 2.7 nm can be prepared via self-assembled method. The TEM results confirm the size and shape of the Damna-NS. The optimum initial drug used in Damna-NS preparation should be 20 % to get encapsulation efficiency of 36.3 %. A bioluminescent yeast-reporter system was used to investigate Damna-NS ’s estrogenic or toxic effects. The initial screening results of the bioluminescent yeast-reporter system revealed that both Damna and Damna-NS had no estrogenic effects on their own, but when treated with a S9 fraction to imitate the metabolism of test chemicals in mammals, they had considerable estrogenic effects, showing that their metabolites are estrogenic. Damna and Damna-NS were found to be harmful in toxicity tests. When compared to the drug alone, the Damna-NS exhibits less toxicity. This suggests that the PhCS-g-mPEG nanosphere may be useful in reducing Damna toxicity. Even after S9 treatment, Damna and Damna-NS showed no toxicity. These findings support the potential benefits of additional Damna-NS research and suggest that Damna-NS could be effective as an oral phytoestrogen in hormone replacement treatment. However, the limitation of this study is that the maximum encapsulation efficiency of Damna-NS is less than 40 % due to Damna's poor aqueous solubility. The physical and chemical properties of drugs can be improved through a variety of methods, including particle size reduction, salt formation, solid dispersion, and use of surfactant. The modified physicochemical properties of Damna would increase the amount of drug entrapped in nanospheres.

## CRediT authorship contribution statement

**Thararat Nualsanit:** Conceptualization, Data curation, Visualization, Writing – review & editing. **Nutsawan Chaisutatip:** Methodology, Data curation, Writing – original draft. **Worapapar Treesuppharat:** Writing – review & editing. **Pleumchitt Rojanapanthu:** Supervision.

## Declaration of Competing Interest

The authors declare that they have no known competing financial interests or personal relationships that could have appeared to influence the work reported in this paper.

## Data Availability

No data was used for the research described in the article.
